# Determining patient eligibility for a physical activity referral scheme through EHR data extraction

**DOI:** 10.1186/s12911-025-03122-4

**Published:** 2025-09-29

**Authors:** James Rosemeyer, Jennifer L. Trilk, Meenu Jindal, John M. Brooks, Lia K. McNulty, Mark Stoutenberg

**Affiliations:** 1 Department of Biomedical Sciences, University of South Carolina School of Medicine Greenville, Greenville, SC USA; 2 Prisma Health, Greenville, SC USA; 3 Department of Health Services, Policy and Management, Arnold School of Public Health, University of South Carolina, Columbia, SC USA; 4 College of Public Health, Temple University, Philadelphia, PA USA; 5 Department of Health Behavior and Nutrition Sciences, College of Health Sciences, University of Delaware, Newark, DE USA; 6 Department of Sport and Exercise Sciences, Durham University, Durham, UK; 7 Wolfson Research Institute for Health and Wellbeing, Durham University, Durham, UK

**Keywords:** Data extraction, Electronic health records, Eligibility, Exclusion criteria, Physical activity, Reach

## Abstract

**Background:**

Physical Activity Referral Schemes (PARS) are an effective treatment option for promoting physical activity and positively impacting patient care. Retrospective evaluation of PARS implementation requires identifying the eligible patient population that was reached. However, during a clinic visit, health care providers (HCPs) may decide that a physical activity referral is not appropriate for various reasons, such as acute illness or recent surgical history. Including patient visits with these health conditions in assessing a PARS may lead to an overestimation of patients eligible for referral.

**Objective:**

To develop a process that more accurately determines patient eligibility for a physical activity referral when retrospectively extracting patient visit data from the electronic health record (EHR).

**Methods:**

Inclusion criteria were developed to identify patient visits potentially eligible for a physical activity referral based on five chronic conditions. These conditions were highlighted during the standardized training that staff received when implementing the PARS. Development of exclusion criteria incorporated exercise contraindications from published literature, input from practicing HCPs, and refinement by a multidisciplinary healthcare team. Inclusion/exclusion criteria were pooled and mapped to International Classification of Diseases, 10th edition, codes and applied to EHR data.

**Results:**

A total of 334 referrals (numerator) were identified from the pool of eligible patient visits meeting the inclusion criteria. In calculating the denominator for our reach estimate, 479,536 patient visits were initially extracted from the EHR. Applying the inclusion criteria, 58% of these visits were PARS-eligible (*n* = 277,515). The eligible visits further decreased by 23% (*n* = 63,203) with the application of the exclusion criteria, leaving a total of 214,312 PARS-eligible visits (denominator), a 55% reduction from the initial number of total patient visits.

**Conclusion:**

Through this multi-step process, we developed a novel approach for retrospectively identifying patient visits eligible for a physical activity referral that can be applied to extracted EHR data for subsequent evaluation. This process can be used by other healthcare systems and researchers in the assessment of PARS. Ongoing refinement of the exclusion criteria is needed to best reflect the eligible population and provide the most accurate estimate of the overall PARS reach.

**Clinical trial registration:**

Not applicable.

**Supplementary Information:**

The online version contains supplementary material available at 10.1186/s12911-025-03122-4.

## Background

It is estimated that 31.3% of adults worldwide were physically inactive in 2022; if this trend continues, inactivity levels are expected to rise to 34.7% by 2030 [1]. While inactivity levels increase, health detriments remain unchanged for those who do not reach physical activity (PA) recommendations [2, 3]. As part of a multi-sectoral approach to address this issue, there is an increasing emphasis on integrating PA interventions in healthcare settings [4]. Physical activity referral schemes (PARS) are multicomponent interventions often implemented in primary care settings [5–9]. Patients engaged in PARS have shown to have improved self-reported PA levels, maintenance of PA, and quality of life compared to those not engaged [5–7]. Key components of PARS may include using a patient-centered approach in conducting PA screening/assessment, providing a written or electronic exercise prescription and/or PA counseling, along with a referral to a PA network [10]. With PARS becoming more common in health settings, evaluating these schemes is necessary to provide guidance on strategies for increasing adoption, enhancing implementation and reach, and informing future healthcare decision-making.

Using purposive electronic health record (EHR) fields or measures (e.g., diagnosis codes, visit type, age), investigators can extract data for a specific patient population to evaluate the *reach* of an intervention, like a PARS. Reach can be described as the number, proportion, and representativeness of individuals willing to participate in a given intervention [11]. More specifically, the reach proportion is typically expressed as the individuals impacted by an intervention (the “numerator”) over the total number of individuals that *could have been* impacted (the “denominator”). During the analysis of an intervention, estimating the numerator requires a proper definition of program engagement, such as the count of individuals receiving a PA referral. Estimating the denominator depends upon the type of intervention being evaluated and the eligible patient population (e.g., individual patient visits, unique patients). In real world situations, estimating the numerator and denominator can be challenging due to operational components, like EHR documentation errors or lack of implementation [12, 13], as well as psychological factors, like the decision-making process of a healthcare provider (HCP) at the point of care.

The point of care decision-making process an HCP undergoes is dynamic with multiple levels of influencing factors, including patient, physician, practice site, organizational, network/hospital, and market environment [14]. Furthermore, a treatment plan becomes more complex as patient co-morbidities increases, requiring the HCP to prioritize certain treatments (e.g., medication adherence) over others (e.g., a PA referral) [15]. With the available information, decisions are made with an underlying acknowledgment that HCPs have different practice styles (e.g., “minimalists”, “interventionists”), resulting in different decisions based on their past education, theories, training, and experiences [16]. Although exercise is a best buy for many chronic conditions [17], there may be several reasons why an HCP may elect not to refer their patients to a PARS. Identifying common factors (e.g., patients with acute illness, recent surgical procedure, etc.) that influence their decision-making process is crucial to avoid overestimating the patient visit population eligible for a referral (the denominator) and underestimating the reach of a PARS.

While PARS have been implemented in numerous different health systems around the world [18–20], most research focuses on individual-level outcomes related to the patients engaging with local PA resources. The goal of this paper is to provide broader guidance to those evaluating the reach of PARS across any health system, regardless of individual differences (e.g., payment models, patient costs, types of health systems). To accurately track the reach of PARS implemented in these health settings, it is essential to develop a refined list of inclusion and exclusion criteria to guide the accurate EHR patient data extraction. The process of identifying these criteria for a population eligible for a PARS has yet to be described. Thus, this article describes the process of identifying and refining eligible patient visits when retrospectively extracting data from EHR records to more accurately assess the reach of a PARS in a major U.S. health system.

## Methods

**Fig. 1:**
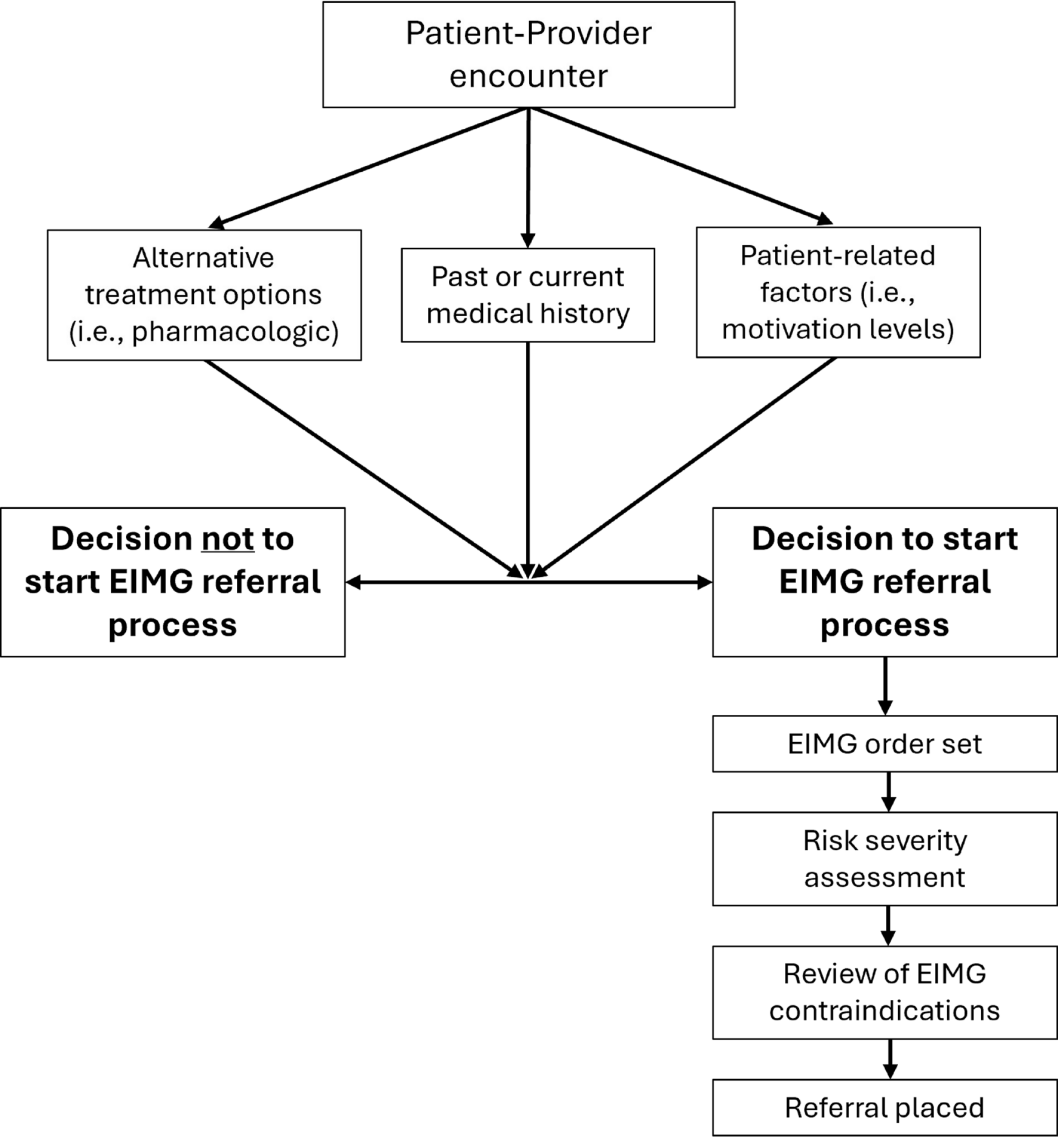
Components of decision-making during a patient-provider encounter, and physical activity referral pathway. EIMG: Exercise is Medicine Greenville^®^

This paper explores the development and implementation of eligibility criteria for a PARS in a large U.S. health system. Exercise is Medicine Greenville^®^ (EIMG) is a comprehensive clinic-to-community PARS integrated in the Prisma Health system in Greenville, South Carolina in 2016 and has since expanded to 37 primary care clinics referring their patients to an evidence-informed, 12-week community-based PA program [21]. Adopting clinics complete a standardized onboard training before referrals can be placed. After consultation with their patient, an HCP places an EIMG referral, connecting the patient to a participating community-based PA facility (see Fig. 1). Patients, 18 to 80 years of age, are eligible for an EIMG referral if they fail to meet weekly PA guidelines [22] or have been diagnosed with, or are at-risk for, a chronic disease (i.e., diabetes, dyslipidemia, hypertension, obesity). At its initiation, the EIMG program developed a list of contraindications to exclude ineligible referred patients from participating in the PA program for safety reasons. A more comprehensive description of the EIMG program and the referral process has been previously described [9].

### Sourcing the data

This paper describes the process of refining an approach to accurately identify eligible patient visits for a PARS. This process was developed using data from a larger study between March 15, 2021 and September 30, 2022, following EIMG reopening due to COVID-19 shutdown in 2020. This research was submitted to and approved by the Prisma Health Institutional Review Board (IRB) Committee A (#1980721), which belongs to the Prisma Health Office of Human Research Protection and conducted in accordance with the Declaration of Helsinki. For the retrospective chart review, collection of data secondary to research, the Prisma Health IRB Committee A (#1980721) approved a waiver of consent in accordance with 45 CFR46.116(f) [3] finding all requirements to be met. The deidentified data transfer was conducted in accordance with relevant guidelines and regulations. The initial dataset included all patients 18–80 years of age who visited 1 of 12 primary care clinics that had previously adopted the EIMG program before the COVID-19 shutdown. The study team extracted diagnosis codes from multiple sources of the EHR, including the ‘problem list’ and ‘encounter diagnosis’ fields.

### Quantifying EIMG referrals

The EIMG referral process is built into the health system EHR, allowing for data extraction of patients referred to the program using the unique EIMG referral code (REF201) and other structured boundaries (e.g., clinic/provider ID, specific dates, etc.). Additional boundaries (inclusion criteria) were placed on EIMG-referred patients that mirrored the chronic conditions (e.g., obesity, hypertension) emphasized with HCPs during standardized onboard training to reflect the target patient population. Documentation of diagnoses in the EHR was completed using the International Classification of Diseases, 10th edition, (ICD-10) Clinical Modification coding system [23]. The aforementioned inclusion criteria health conditions were mapped to ICD-10 codes and applied to the list of EIMG-referred patients.

### Identification of eligible patient visits

Patient ‘visits’ were chosen, instead of ‘unique patients’ as the unit of measurement for the assessment of referral rate, as each visit represents a unique opportunity for patients to receive an EIMG referral. Over the continuum of care, a referral may be more appropriate at one visit versus another. In estimating the total number of potential visits in which patients would have been eligible to receive an EIMG referral based on data extracted from the EHR, the first step involved identifying all patient visits eligible for an EIMG referral using the inclusion criteria described in *Quantifying EIMG Referrals* above.

### Potential exclusion criteria

Next, the list of potentially eligible patient visits was refined by removing visits with patients unlikely to have been considered for a referral by their HCP using a three-step process.

#### Review of published literature

First, existing guidelines listing contraindications for exercise participation were reviewed. The American College of Sports Medicine (ACSM) Guidelines for Exercise Testing and Prescription (GETP), 10th edition, specifically the algorithm in Chap. 2 (Exercise Preparticipation Health Screening) and the Physical Activity Readiness Questionnaire for Everyone (PAR-Q+) were reviewed for exclusionary criteria [24, 25]. The PAR-Q + is designed for the lay person to determine whether they can participate in PA safely without first seeing an HCP for clearance.

#### Expert recommendations

Three local HCPs (a family medicine physician, a family medicine physician certified in lifestyle medicine, and a bariatric surgeon) familiar with EIMG and four external HCPs (three primary care sports medicine physicians, and a physician dual board-certified in sports medicine and physical medicine & rehabilitation) with current or past affiliation with the ACSM’s Exercise is Medicine^®^ initiative were contacted individually by email and asked to provide potential indications that would preclude them from providing a patient with a PA referral.

#### Multidisciplinary team meetings and ICD-10 mapping

Potential exclusionary health conditions from existing guidelines were pooled with the indications listed by the HCP expert recommendations. All items were then discussed by a multidisciplinary clinical team consisting of a primary care physician, registered nurse, and two board-certified lifestyle medicine experts. Additionally, a health economist provided high-level insight into coding nuances (i.e., collapsing and grouping codes) and other systems used (i.e., Charlson Comorbidity Index) from their experienced past with large EHR datasets. Group consensus was required before items were removed or added to a final list of exclusionary health conditions. The refined list of health conditions was mapped onto all associated ICD-10 codes by a member of the research team. Codes were identified using a World Health Organization online resource [26] and the health system EHR system. The code mapping created an expanded list (e.g., the condition “chronic kidney disease” maps to seven distinct ICD-10 codes) and a second multidisciplinary team meeting established a final list of exclusion codes.

### Grouping and truncating codes

Due to the extensive detail in the ICD-10 Clinical Management coding system, some codes were grouped together for ease of future data extraction and analysis. For example, asthma can be identified by 18 unique ICD-10 codes. Moderate and severe asthma, identified as exclusion criteria for EIMG, had their codes simplified into two truncated codes. Asterisks at the end of the code were used as ‘wildcards’, allowing for multiple codes to be identified with different ending characters (see Table 1).

**Table 1:** Grouping and truncating ICD-10 codes

ICD-10 code	Description	Modified code
J45.20	Mild intermittent asthma, uncomplicated	n/a
J45.21	Mild intermittent asthma with (acute) exacerbation	n/a
J45.22	Mild intermittent asthma with status asthmaticus	n/a
J45.30	Mild persistent asthma, uncomplicated	n/a
J45.31	Mild persistent asthma with (acute) exacerbation	n/a
J45.32	Mild persistent asthma with status asthmaticus	n/a
J45.40	Moderate persistent asthma, uncomplicated	J45.4*
J45.41	Moderate persistent asthma with (acute) exacerbation	
J45.42	Moderate persistent asthma with status asthmaticus	
J45.50	Severe persistent asthma, uncomplicated	J45.5*
J45.51	Severe persistent asthma with (acute) exacerbation	
J45.52	Severe persistent asthma with status asthmaticus	
J45.901	Unspecified asthma with (acute) exacerbation	n/a
J45.902	Unspecified asthma with status asthmaticus	n/a
J45.909	Unspecified asthma, uncomplicated	n/a
J45.990	Exercise induced bronchospasm	n/a
J45.991	Cough variant asthma	n/a
J45.998	Other asthma	n/a

Individual diagnoses with linked ICD-10 code associated with the condition of asthma. n/a: code was not selected as an exclusion criteria diagnosis

## Results

### EIMG referred patients

**Fig. 2:**
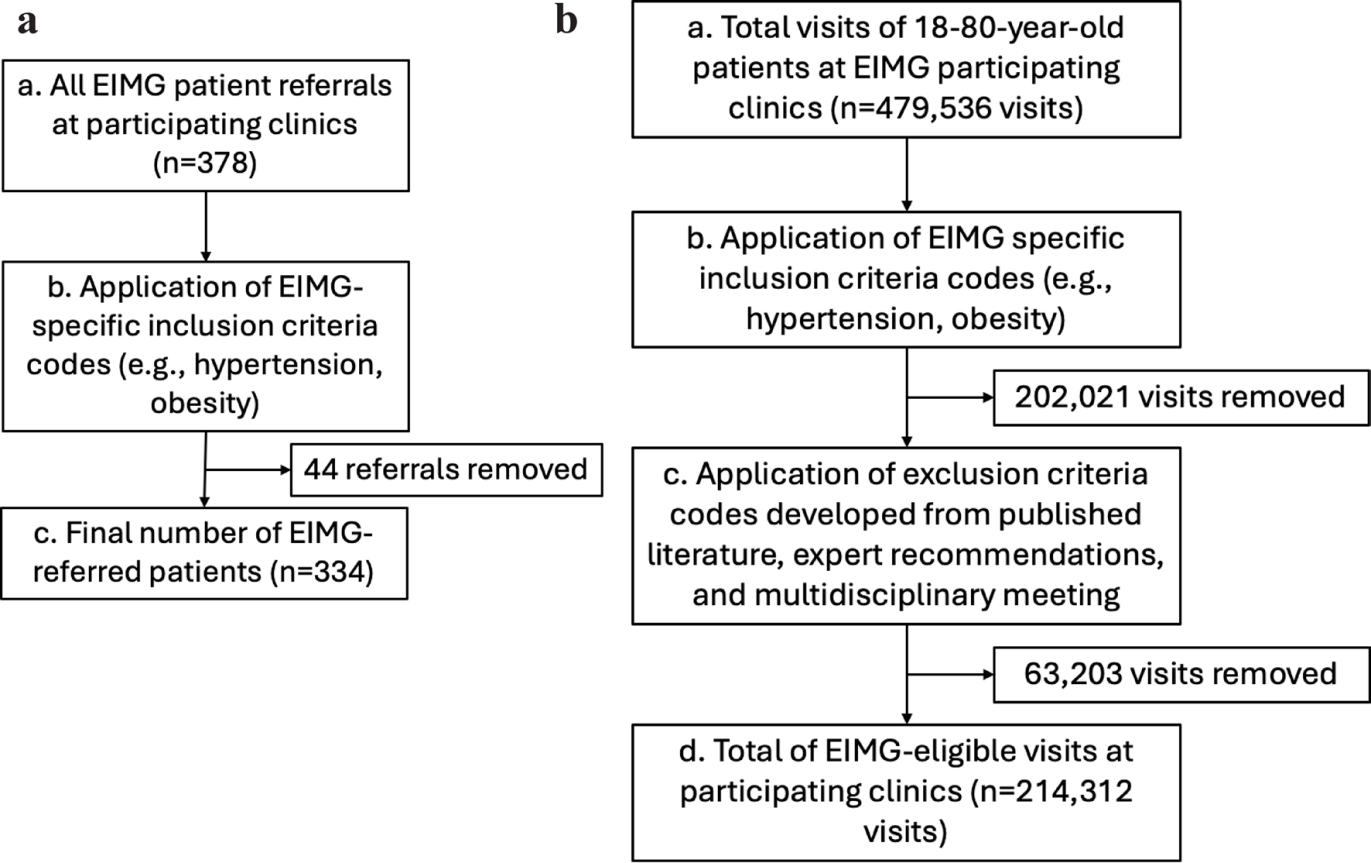
**a**. Identifying EIMG-referred patients (numerator). **b** Refining the patient population potentially eligible for an EIMG referral (denominator)

The five inclusion criteria (physical inactivity, obesity, dyslipidemia, diabetes, and hypertension) were mapped to 30 unique ICD-10 codes (see Additional file 1). Three unique codes (exercise counseling, lack of physical exercise, and other specified personal risk factors [e.g., sedentary]) were identified for the condition of physical inactivity. Seventeen codes were associated to obesity with the majority describing levels of body mass index (BMI). Dyslipidemia was identified by four unique codes (hyperlipidemia, mixed hyperlipidemia, pure hyperglyceridemia, and pure hypercholesterolemia), five codes were linked to diabetes, and one was linked to hypertension. Applying these diagnosis codes to the dataset of all EIMG-referred patients (Fig. 2a; box b), resulted in the identification of 334 EIMG referrals, representing the numerator in estimating the proportion of the eligible patient population reached (Fig. 2a; box c).

### Patient population and inclusion of eligible visits

There were 479,536 total visits (Fig. 2b, box a) at EIMG-activated primary care clinics during the study period. After applying the inclusion criteria (Fig. 2b, box b), 202,021 visits were removed (42% reduction), resulting in 277,515 EIMG-eligible visits.

### PARS exclusion criteria

Review of the ACSM GETP and the PAR-Q + resulted in the identification of 16 and 24 broad items, respectively, that may preclude an individual from participating in a structured PA program. Some items described definitive health conditions (e.g., recent myocardial infarction), while others, like dizziness, corresponded to more than one health condition (e.g., embolism, atrial fibrillation). Seven practicing HCPs identified 19 unique items that may preclude an individual from receiving a referral to a structured PA program. Some responses were broad (e.g., severe musculoskeletal pain/injuries), but were counted as only one unique item with the intent to further define the item through the collaborative meetings. Some items addressed a situation (e.g., patients qualifying for cardiac rehab) instead of a specific health condition and again were included as one item to be clarified in future meetings. The breakdown of identified conditions and their groupings can be found in ‘Additional file 2’.

Thirty-seven unique items were compiled and shared with the multidisciplinary team. After discussion, four of the items (coronary artery disease, diabetes, osteoporosis, back problems/acute back injury) were removed as it was determined that these should not be considered as contraindications for structured PA. The low threshold of the ACSM GETP and PAR-Q + to refer an individual to an HCP for PA clearance was reason for these conditions being identified in the initial search but ultimately being removed. In defining the broad or situational responses, more health conditions were added to the exclusion criteria, including embolism, aortic stenosis, atrial fibrillation, and fractures. A member of the research team mapped all exclusionary items onto all eligible ICD-10 codes. The process of mapping often expanded one condition to multiple ICD-10 codes, describing different disease pathologies or severity (e.g., acute versus chronic, disease stages). This expanded list of codes was discussed a second time with the multidisciplinary team to establish a final list of 78 truncated exclusion codes (see Additional file 3).

The application of these exclusion criteria (Fig. 2b; box c) decreased the EIMG-eligible visits to a final total of 214,312 visits that were likely appropriate for an EIMG referral (Fig. 2b, box d), a 55% reduction from the initial total patient visits (479,536) extracted from the EHR.

## Discussion

A major challenge when evaluating the reach of a PARS is that referrals are often initiated by HCPs based on their perceptions of patient priorities and needs. The more we understand which patients may or may not be targeted for a referral by HCPs, the better we can assess the true reach and impact of PARS and other healthcare innovations. This article is the first to describe the process of refining a target population of patients and their potentially eligible visits to receive a PA referral based on EHR data. Our process of developing inclusion and exclusion criteria to extract EHR patient data specific to receiving a PARS referral increased the accuracy of the data analysis and resulted in a more precise estimate of overall program reach.

The reach of PARS using a denominator consisting solely of ‘total patient visits’ does not accurately reflect the variability in HCP PA promotion for a particular patient at a given time [27]. Identifying exclusionary criteria is a challenging task when considering the various educational and training backgrounds and personal experiences of each HCP underlying their decision-making process when treating a patient. As a result of this work, we developed a list of 30 inclusion and 78 exclusion criteria ICD-10 codes that were applied to extracted EHR data for assessment of PARS referral rates. In our sample, 55% of the initial 479,536 visits were removed after applying both the inclusion and exclusion criteria.

The importance of identifying accurate target populations has been described in the assessment of national immunization programs [28], as well as in the development of quality measures for hospital systems [29, 30]. When evaluating a colorectal cancer screening intervention that identified eligible patients with a daily-updated EHR registry, Green et al. [13] found they were able to more accurately show intervention reach when accounting for factors, such as EHR system and clinic delays in intervention initiation. In the context of palliative care and surgery, Lee et al. [30] worked to define the term serious illness in order to create a denominator for quality measure efforts. Similarly, we looked to more accurately define the denominator by identifying factors contributing to the point-of-care interactions leading to potential PA referral. For example, a provider treating acute illness in a patient with prediabetes is unlikely to refer the patient to a PARS at that particular encounter. By applying the acute illness diagnosis code as exclusion criteria, that encounter can be retroactively removed from the denominator.

The process used to identify inclusion/exclusion in this study can be used by other health service researchers when evaluating PARS using retrospective data extraction from EHRs. While the inclusion criteria were developed specifically for the target population of the EIMG program, these criteria may be adapted to include patients of different ages (e.g., older adults) or health conditions (e.g., depression/anxiety) that could benefit from a PA referral to increase PA behaviors. The exclusion criteria list can be similarly modified for use with any PARS with a similar target population through tailoring of diagnosis codes where appropriate. When refining the inclusion/exclusion criteria for a data sample from an EHR, it is essential to involve a range of key stakeholders, including experts in the field, clinicians, clinic managers and other leaders in the health care system. The ICD-10 Clinical Management coding system used in this study is recognized by all major U.S. health systems, allowing for broad generalizability. For health settings outside of the U.S., use of this work may require verification of the specific codes against their country’s own ICD-10-modified coding system. Finally, consideration should be given to the inclusion/exclusion lists that further refinement will be required as the 11th edition of the ICD coding system becomes adopted worldwide.

There are several limitations with this work. The accuracy of retrospectively identifying patients with a marked diagnosis code is dependent on proper EHR documentation. In some instances, health conditions discussed during an encounter and documented in the HCP’s note may not be recorded as frequently in the diagnosis field area of the EHR [31]. Second, while we attempted to be exhaustive in our efforts, there are likely additional, less frequently used codes that were not captured in the exclusion list. The size and complexity of the ICD-10 Clinical Management coding system (> 65,000 individual codes) makes accounting for these omissions an imperfect science, requiring ongoing refinement for those utilizing these criteria. Thirdly, identifying exclusionary health conditions for PA referral that are universally adopted by HCPs is challenging. While the initial list of excluding conditions began with input from multiple HCPs, each HCP uniquely approaches patient care (e.g., giving a PA referral) and it is not possible to predict their decision-making process with certainty. Further work is needed in refining the list of diagnoses to exclude a patient from PA referral and future research should test the validity of these methods versus a sample of true patient encounters.

At the same time, our study has a number of strengths that increase the validity and strengthen the external generalizability of our work. We employed a rigorous process in developing the inclusion/exclusion criteria in a real-world application, not a hypothetical simulation. Another strength is that this work incorporated scientific guidelines and multiple key stakeholders with direct knowledge and experience with PARS.

### Examining reach by unique patients

While our analysis prioritized patient visits, a similar set of principles can be applied to examining reach by unique patients with a few additional considerations. Using the same data set as previously presented, we started with 110,362 potentially eligible unique patients. This was reduced to 66,993 and then 60,102 unique patients (a 46% reduction from the initial unique patient population) after applying the inclusion and exclusion criteria, respectively. It is important to note several challenges that come with identify patients during EHR data extraction. A data extraction request for ‘unique patients’ can result in different totals (e.g., denominator) depending on the method used. For example, if a patient was treated at one clinic before switching care to another clinic during the designated time period, they will count as a unique patient for each clinic when aggregating totals across individual clinics. However, as we did in our analysis, if the query is for unique patients across all study clinics, the same patient will only be counted once. Similarly, patients that see multiple providers at a clinic may result in being double counted when assessing unique patients at the clinic level. Thus, it is important to clearly identify and define data fields to ensure consistent extraction from the EHR.

## Conclusion

This study is the first to describe the process of identifying key inclusion and exclusion criteria that can be used to more accurately represent the target population of patient visits when evaluating a PARS. To date, no previously published work has provided such guidance and examples on more accurately defining the target patient population. Applying this process to extracted EHR patient data refines the eligible population of patient visits, providing a better understanding of the reach and overall impact of a PARS. This work can be directly implemented by other U.S. health service researchers when evaluating their PARS, while other countries using a different ICD-10 modification may need to verify code accuracy before proceeding. Further work is needed for ongoing refinement of the exclusion criteria to reflect the most accurate population eligible for a PA referral.

## Electronic supplementary material

Below is the link to the electronic supplementary material.

Supplementary Material 1: Additional file 1: Inclusion criteria codes. .xlsx file with ICD-10 diagnosis codes and descriptions for the identified inclusion criteria in this project.

Supplementary Material 2: Additional file 2: Potential exclusion conditions, grouped by source. .xlsx file with potential excluding health conditions for physical activity referral, grouped into three sources.

Supplementary Material 3: Additional file 3: Exclusion criteria codes. .xlsx file with ICD-10 diagnosis codes and descriptions for the identified exclusion criteria in this project.

## Data Availability

The data that support the findings of this paper came from a larger study and are available upon request from the corresponding author. The data is not openly shared due to Prisma Health data sharing policies.

## Declarations

### Relevant guidelines and regulations

All methods of data handling were conducted in accordance with relevant guidelines and regulations through the Prisma Health IRB Committee A (#1980721).

## Abbreviations

ACSM: American College of Sports Medicine
EHR: Electronic health record
EIMG: Exercise is Medicine Greenville^®^
HCP: Healthcare provider
ICD: International classification of diseases
PA: Physical activity
PAR-Q +: Physical Activity Readiness Questionnaire for Everyone
PARS: Physical activity referral scheme
U.S.: United States
